# Thyroid Cancer Diagnostics Related to Occupational and Environmental Risk Factors: An Integrated Risk Assessment Approach

**DOI:** 10.3390/diagnostics12020318

**Published:** 2022-01-27

**Authors:** Gabriela Maria Berinde, Andreea Iulia Socaciu, Mihai Adrian Socaciu, Andreea Cozma, Armand Gabriel Rajnoveanu, Gabriel Emil Petre, Doina Piciu

**Affiliations:** 1 Department of Occupational Health, University of Medicine and Pharmacy “Iuliu Hațieganu”, 400012 Cluj-Napoca, Romania; gabriela_berinde@yahoo.com (G.M.B.); armand.rajnoveanu@umfcluj.ro (A.G.R.); 2 Department of Radiology and Imaging, University of Medicine and Pharmacy “Iuliu Hațieganu”, 400012 Cluj-Napoca, Romania; mihai.socaciu@umfcluj.ro; 3 Department of Endocrinology, Medisyn Medical Clinic, 400474 Cluj-Napoca, Romania; ada_cozma@yahoo.com; 4 Department of Surgery 4, University of Medicine and Pharmacy “Iuliu Hațieganu”, 400012 Cluj-Napoca, Romania; dr_gabipetre@yahoo.com; 5 Doctoral School, University of Medicine and Pharmacy “Iuliu Hațieganu”, 400012 Cluj-Napoca, Romania; doina.piciu@gmail.com

**Keywords:** thyroid cancer diagnosis, occupational risk, environmental risk factors, oncometabolites, risk assessment matrix

## Abstract

There are still many questions remaining about the etiopathogenesis of thyroid cancer, the most common type of endocrine neoplasia. Numerous occupational and environmental exposures have been shown to represent important risk factors that increase its incidence. Updated information about thyroid cancer diagnostics related to occupational and environmental risk factors is reviewed here, considering an integrated risk assessment approach; new data concerning thyroid cancer etiology and pathogenesis mechanisms, diagnostic biomarkers and methodologies, and risk factors involved in its pathogenesis are presented. A special emphasis is dedicated to specific occupational risk factors and to the association between environmental risk agents and thyroid cancer development. The occupational environment is taken into consideration, i.e., the current workplace and previous jobs, as well as data regarding risk factors, e.g., age, gender, family history, lifestyle, use of chemicals, or radiation exposure outside the workplace. Finally, an integrative approach is presented, underlying the need for an accurate Risk Assessment Matrix based on a systematic questionnaire. We propose a complex experimental design that contains different inclusion and exclusion criteria for patient groups, detailed working protocols for achieving coherent and sustainable, well-defined research stages from sample collection to the identification of biomarkers, with correlations between specific oncometabolites integrated into the Risk Assessment Matrix.

## 1. Introduction

Cancer is a global challenge that has a significant impact on human mortality and morbidity, as well as an increasing incidence worldwide. A special place in current research is represented by the discovery and study of tumor etiology, pathogenesis, and biomarkers that can diagnose and predict the evolution of malignancy. Moreover, numerous occupational and environmental exposures have been shown to represent important risk factors that increase the incidence of thyroid cancer (TC). TC, the most common type of endocrine neoplasia, is still among those with the least amount of information available concerning the etiopathogenesis of all its cancerous forms. 

Numerous occupational and environmental exposures have been shown to disrupt endocrine function, but much less is known about their relationship with TC [1]. 

The number of new cases is constantly increasing, with TC ranking ninth in the top types of cancer mentioned by Globocan 2020 [2]; therefore, retrospective and prospective studies are welcome. 

## 2. Etiology and Pathogenesis Mechanisms

Thyroid masses are often diagnosed in the general population as palpable nodules or during routine ultrasound examinations. The vast majority are benign, but they require active surveillance. Five to ten percent of all thyroid nodules are malignant. There are several types of TC that can be classified, based on their morphological characteristics, as differentiated (papillary and follicular) and undifferentiated (medullary, anaplastic) carcinomas. Papillary carcinoma is the most common type, affecting 80% of patients; follicular carcinoma is more common in older subjects and accounts for about 10% of cases; medullary carcinoma accounts for about 3% of all cases; anaplastic carcinoma is the least common and the most deadly, with a low survival rate [3].

Papillary carcinoma (PTC) is diagnosed frequently between the ages of 30 to 50, with a very good prognosis for early diagnosis. About 43% of all TC and 50% of new cases are papillary microcarcinoma (nodules measuring less than or equal to 1 cm), a subtype of papillary carcinoma. Follicular carcinoma (FTC), the second most common type, is more likely to spread to other organs such as the lungs and bones than the papillary subtype. Medullary carcinoma is less differentiated than the papillary and follicular types and is frequently accountable for high levels of calcitonin and carcinoembryonic antigen, with a higher risk of metastasis than the follicular subtype. Anaplastic carcinoma is the most undifferentiated type, accountable for 2% of all cases; it is a very aggressive form of cancer, and spreads quickly in the neck and to distant organs. Other types of undifferentiated carcinoma include thyroid lymphoma, squamous cell carcinoma, sarcoma, or Hürtle cell carcinoma [3,4]. 

Despite research efforts, current knowledge on the etiology of thyroid carcinoma remains limited. Advances in the elucidation of the pathogenesis of TC includes genetic and proteomic investigations. A common germline polymorphism of the p53 gene was found to produce an arginine to proline change in amino acid position 72 [5]. The resulting codon 72 variants have been reported, being associated with tumor susceptibility, reducing p53’s ability to activate apoptosis. This codon 72 polymorphism reduces vulnerability to carcinogens and may explain the ethnic variations in TC frequency. In this study, data on lifetime occupational history, smoking history, general health conditions, previous diseases, and other anamnestic data was also obtained through interviews. Patients with TC showed a significant overrepresentation of codon 72 variants compared to the control population. The Pro/Pro genotype, after adjusting for gender, age, tobacco, and drug consumption, was associated with a markedly higher risk of FTC, confirming that p53 polymorphism is implicated in thyroid carcinogenesis and that individuals harboring the Proline/Proline genotype have an increased risk of developing TC [5]. A recent study investigated the potential effect of protein 53 (p53) reactivation by the peptide heterodimer Pep3 and its impact on the immune response in robust TC-presenting autoimmunity. In the peripheral blood, Pep3 treatment alters the percentages of CD8+ and CD4+ T regulatory and effector cells of TC patients, favoring an anticancer immune response [6]. The BRAF gene mutations represent a novel indicator of the progression and aggressiveness of thyroid carcinogenesis. In another recent study, the involvement of BRAF gene mutations and their expression in North India patients, as well as their association with pathological characteristics, was investigated. Using PCR followed by DNA sequencing, and evaluating BRAF protein expression by Western Blotting, BRAF mutations affecting codon 600 (valine to glutamine) were identified, restricted only to PTC. Patients with well-differentiated disease and elevated thyroid-stimulating hormone levels were significantly associated with BRAF mutations. Overall, 90% of TC cases showed increased expression of BRAF, and non-smokers were significantly associated with BRAF over-expression. Therefore, both mutational events and over-expression of the BRAF gene are highly implicated in TC pathogenesis and BRAF protein overexpression is independent of the BRAF mutational status of TC patients [7,8,9].

The polymorphism in genes encoding for enzymes involved in the biotransformation of carcinogens is relevant as a risk factor for TC and may be of considerable importance from a public health point of view. N-acetyltransferase 2 (NAT2) polymorphism modulates the response to ionizing radiation, the strongest risk factor recognized as causing differentiated TC thus far; therefore, the influence of the NAT2 detoxification system on TC susceptibility is important.

A prospective case–control study compared 195 TC patients, including 164 PTC and 31 FTC, with 196 control individuals paired for gender, age, ethnicity, diet, lifetime occupation, smoking status, general health, and medical history. The PCR-RFLP assays and the sequencing of six variant alleles determined 18 NAT2 haplotypes that defined different phenotypes, such as slow, intermediate, and rapid—some of which were identified as risk factors. No association was found between genotype and the patient’s clinical or pathological status or their laboratory results, regarding genotypes and outcome, which suggests that NAT2 detoxification is involved in TC pathogenesis [10].

Regarding tobacco users, many epidemiological studies have reported a reduced risk of differentiated TC for this category, in opposition to most human carcinomas. The 1A1 gene variants of cytochrome P450 are associated with an increased ability to activate polycyclic aromatic hydrocarbons, resulting in highly reactive electrophilic intermediates which may result in DNA damage. Consequently, inheriting a germline type mutation of the CYP1A1 gene may decrease the susceptibility for TC. One older study [11] was designed to investigate the role of Cytochrome P450 1A1 in thyroid carcinogenesis and its possible association with codon 72 of the p53 genotypes. This case–control study had a total of 248 patients with thyroid pathology and 277 controls with similar ethnic backgrounds. In the patient group, there were included 67 cases of benign goiters, 13 patients with follicular adenomas, 136 diagnosed with PTC, and 32 with FTC. All participants were interviewed regarding their dietary and smoking habits, occupational history, and illness history. By PCR analysis, it was found that the wild-type genotype of Cytochrome P450, 1A1, was more frequently identified in PTC patients (74.26%) than in the control group (62.45%), which signifies a reduced risk for this type of cancer. The statistical analysis, using a multiple logistic regression model, revealed an inverse correlation between inherited germline CYP1A1 (susceptibility to papillary carcinomas) and smoking. They found no relations between smoking habits, clinical findings, or different types of parameters that are linked to aggressiveness at diagnosis. 

A study highlighted the association between thyroid disease and autoimmune skin disease (AISD) through their shared susceptibility genes and antigens, linked to the expression of thyroid hormone receptors and thyroid hormone deiodinases. Candidate gene association studies and genome-wide linkage analysis identified nine loci involved in both vitiligo and autoimmune thyroid disease, including genes coding for tyrosine (TYR), thyroglobulin (Tg), and thyroid-stimulating hormone receptor (TSHR). The reported evidence showing a relationship between dysregulated TSH, thyroid hormone action, and AISD warrant further investigations, including their role in carcinogenesis [12].

## 3. Thyroid Cancer Diagnostics: General Approach

Thyroid nodules (TNs) are discreet lesions of the thyroid parenchyma, radiologically distinct from the adjacent tissue [13]. For known or suspected TNs, or those detected as incidental imaging findings, the initial management plan includes determination of thyroid-stimulating hormone (TSH) levels and ultrasound of the thyroid and neck. If TNs are associated with a low TSH level, the function of the nodular tissue must be evaluated using radioiodine imaging, ^123^I thyroid uptake, and a scan. In case of hypofunctional low TSH nodule(s) and for nodule(s) associated with normal or high TSH levels, the ultrasound-guided FNA is advised, also taking into consideration clinical and sonographic features [14]. 

### 3.1. Ultrasonography Diagnosis (US) and Risk Stratification Systems

Ultrasonography (US) is the imaging method of choice for initial diagnosis, due to its high availability, great resolution, and multimodal approach [15]. 

“Incidentally discovered nodules” are nonpalpable nodules frequently found by US only; however, their risk of malignancy is not lower than in the case of palpable lesions. On the other hand, the vast majority of US-discovered nodules prove to be benign, which calls for a robust strategy of risk stratification [13,16]. 

Several US features are associated with malignancy, such as hypoechogenicity, irregular margins, “taller-than-wide” shape or microcalcifications, but none of them alone is sufficient to detect thyroid cancer accurately. Thyroid nodules with suspicious features are often biopsied using ultrasound-guided fine needle aspiration (FNA) [17]. Typically, only nodules larger than 1 cm are concerning, although nodules measuring less than 1 cm may require further investigation when they are associated with clinical symptoms or lymphadenopathy. In very rare cases, some nodules less than 1 cm in size may not show US or clinical warning signs, but nevertheless may cause morbidity and mortality in the future, requiring subsequent follow-up [14].

There is currently no globally accepted risk stratification system for TN. The most recent proposed scores are the American College of Radiology (ACR) TI-RADS (2017) [18], European (EU) TI-RADS (2017) [19], and Korean (K) TI-RADS (2016) [20]. They all share similar concepts of risk stratification, by grouping nodules by their radiological features into five categories (the higher the category number, the higher the risk of malignancy). A comparison between these systems can be found in Table 1.

The main aim of TI-RADS systems is to define US characteristics of nodules requiring further imaging assessment or FNA. 

The vascularity of TN is usually observed by power Doppler US or color DopplerUS and can show three main patterns [19]: absence of intranodular or perinodular flow (Type 1); presence of perinodular and/or slight intranodular flow (Type 2); and presence of marked intranodular and slight perinodular flow (Type 3). Benign lesions are most commonly associated with types 1 and 2 and malignant lesions with type 3, but the sensitivity and specificity are relatively low [21]. 

US elastography can non-invasively assess the TN stiffness, either by data obtained from color maps or by the measurement of “share wave” velocities (SWE, expressed in kPa or as m/s). SWE seems to have the potential to exclude malignant nodules, which should show a higher stiffness, but the required cut-off values still range widely between 35–90 kPa across current studies [22]. 

Neither Doppler US, nor elastography are included in the TI-RADS systems, but they can be useful as complementary features in borderline cases [19].

### 3.2. US-Guided Fine Needle Aspiration (FNA)

US alone cannot diagnose malignancy with sufficient sensitivity and specificity, so it is routinely followed by FNA in suspicious cases. Since a lot of suspicious TNs still prove to be benign, the main role of FNA is to rule out malignant tumors requiring surgery, thus reducing the number of unnecessary surgeries in patients with thyroid nodules. 

The Bethesda System for Reporting Thyroid Cytopathology (BRSTC) is a diagnostic classification system based on FNA cytology. It comprises six categories: non-diagnostic or unsatisfactory (1), benign (2), atypia of undetermined significance (AUS) or follicular lesion of undetermined significance (FLUS; 3), follicular neoplasm or suspicious for a follicular neoplasm (4), suspicious for malignancy (5), and malignant (6) [23].

Although FNA is associated with high diagnostic accuracy and safety, it has limitations due to the risk of sampling error (especially in very large nodules) and a substantial rate of inconclusive results (approximately 20%), including category 3 (AUS/FLUS) [24,25]. In such cases, it is recommended to repeat FNA at 6 months [26], perform short-interval US follow-ups (at 3 month), or even refer to surgery. Some of the shortcomings of FNA can be removed by using core needle biopsy (CNB) for a more reliable histopathology diagnosis and a larger sample, in exchange for a marginally higher risk [27]. 

## 4. Thyroid Cancer Diagnostics: Biomarkers and Methodology

The current diagnosis and classification tools for TC include ultrasound examination, fine-needle aspiration (FNA), and histological examination following thyroidectomy. The prognostic is very different according to the histological subtype, but, in general, the mortality rate for patients with TC is lower with early diagnosis [28]. Fine needle aspiration biopsy is the current gold standard for the diagnosis of TC, but this technique has been shown to give uncertain results and may be unable to discriminate between various carcinomas, thus requiring additional procedures to obtain a final diagnosis [29]. The fine needle aspiration technique reduces unnecessary thyroid surgery by approx. 25%; however, the prevalence of non-diagnosis is still high—approx. 30% [30,31]. Therefore, it is necessary to find reliable markers to complete and improve current methods. Early detection of TC is one of the “success keys” in reducing mortality.

Tumor biomarkers may be nonspecific (found in several types of cancer) or specific to a particular type of cancer, having different profiles in comparison to normal tissues or healthy people. Generally, tumor biomarkers are assessed as biomolecules (DNA and RNAs, proteins and peptides, non-protein metabolites) in cells, tissues, or in biofluids (blood, urine, saliva, etc.).

TC biomarkers may show differences between groups of patients with benign and malignant pathologies and can help identify risks regarding occupational and environmental exposures, lifestyle, diet, age, gender, etc. Consequently, the study of TC tumor biomarkers correlated with different risk factors is of great scientific and therapeutic interest. However, there are still no reliable specific biomarkers for the detection and staging of TC. 

Modern technologies based on molecular sciences, generically called “omics”, address distinct directions of diagnosis and investigation, from genomics to proteomics and metabolomics. Genomic studies reflect the genes involved in predicting this type of cancer, elucidating mechanisms of pathogenesis, while proteins and different classes of metabolites (small molecules with molecular mass less than 1500 Da) can reflect “downstream” activity at the cellular level and the consequences of the tumor development cascade. Tissue proteomic analyses for TC and benign follicular lesions (adenoma) have revealed the involvement of certain proteins (free or associated with lipid metabolism). In addition to the ability to discriminate between cancers, their integration with transcriptomic and metabolomic data allows a more complete understanding of the pathogenesis of these cancers. In comparison with proteomics and transcriptomics, there are fewer studies to date regarding the applications of metabolomics in thyroid cancer.

The methodology for an accurate molecular diagnosis includes PCR (polymerase chain reaction for genomic analysis), MALDI-TOF MS (proteomic analysis), and chromatography (high-performance gas or liquid chromatography) coupled with mass spectrometry (GC–MS and LC–MS) or NMR for metabolite separation and identification. Such techniques offer accurate data which may establish the specific genome, proteome, and metabolic profile of TC [29,32,33,34,35,36,37,38].

The integration of multiple “omics” approaches can provide a more holistic understanding of TC and confirm prospects for clinical applications. In the past, much of the assessment of metabolic changes in the development of TC has been limited to measuring individual hormone and metabolite levels using imaging techniques or standardized laboratory tests. As a more accurate technique, metabolomics is designed for systematic measurements of a variety of metabolites or small molecules such as vitamins, minerals or other nutrients, drugs, and mediators in tissue samples, blood, urine, or any other body fluid [36]. 

Alterations in thyroid cell metabolism reflected in biofluids (blood and urine) can be investigated by new approaches using omics technologies—particularly proteomics and metabolomics. 

Specific proteomic techniques such as MALDI-TOF MS can identify peptides, small proteins (<20,000 Da), and certain lipid biomarkers. The studies mentioned below emphasize the intensification of lipid biosynthesis through the lipidomic profile, highlighting the accentuated tissue biosynthesis of phospholipids such as phosphatidylcholine (16:0/18:1) and (16:0/18:2) [32,39].

The identification of oncometabolites is an important contribution to the establishment of current TC diagnosis and management protocols [40,41]. Metabolomics is a powerful tool that can identify cancer biomarkers and drivers of tumorigenesis and open up future opportunities to improve diagnosis, monitoring, and treatment [42].

A systematic search of MEDLINE and Web of Science databases, with 31 studies investigating metabolite biomarkers of TC, was performed in a recent review which showed specific metabolite alterations within the plasma, FNA specimens, and tissue of TC vs. benign samples in healthy subjects [43]. 

Another complex study on the applications of metabolomics in TC research highlighted the most important steps required for untargeted (metabolic profile) and targeted analysis (targeted to certain well-specified metabolites, or those highlighted in previous studies) using specific LC–MS techniques (liquid chromatography coupled with mass spectrometry) [29,34].

Demonstration of the presence of tissue oncometabolites specific to TC, such as high levels of lactate and choline and low levels of citrate, glutamine, and glutamate, have been reported as biomarkers for determining the metabolomic profile of preoperative thyroid nodules. Tissue lipids including cholesterol, choline, and phosphocholine have shown significant changes in tumor tissues compared to healthy tissue [44]. It has also been found that de novo synthesis of fatty acids is associated with tumorigenesis. Elevated concentrations of methionine, leucine, tyrosine, and lysine—as well as lipids (especially phospholipids and sphingolipids)—have been identified in plasma and PTC tissues [37]. In previous studies, the metabolites proposed by HPLC–MS analysis as biomarkers of TC tissue have included glucose, galactose, lactate, inositol, hypoxanthine, citrate, cholesterol, and choline [35,38].

A recent research work explored the relationship between the tissue redox status and the antioxidative capacity of metal cofactors and their influence on the clinical outcome for PTC and colloid goiter (CG). The study group included 65 PTC and 45 CG. For the redox status, the antioxidant mechanisms were assessed through the activity of glutathione peroxidase (GPx), glutathione reductase (GR), catalase (CAT), and superoxide dismutase (SOD), and the pro-oxidation mechanisms by measuring the malondialdehyde (MDA) concentration levels. For the antioxidative effect of cofactor metals, the levels of Se, Cu, Zn, and Mn were measured. According to the results, PC tissues had an increased GR and GPx activity, but normal pro-oxidative levels. SOD was increased in smokers and showed significantly reduced activity in multicentric PTC dissemination, its activity being directly dependent on the MDA levels of tissues from CG patients. Patients with retrosternal CG had reduced SOD activity and MDA levels. There were many correlations between redox parameters in the PTC tissues, which showed a cooperative response on pro-oxidation and a good co-activation of antioxidative mechanisms. In comparison to other tissues, the PTC tissues had low Se and high Cu and Mn levels. SOD activity and MDA levels were significant predictive factors for lymph node metastases and multicentric dissemination of PTC. The level of Cu predicted the retrosternal localization of CG. This research presented results that provided a significant database for the development of new prognostic biomarkers for PTC and CG [44].

## 5. Risk Factors Involved in the Pathogenesis of Thyroid Cancer

**Figure 1:**
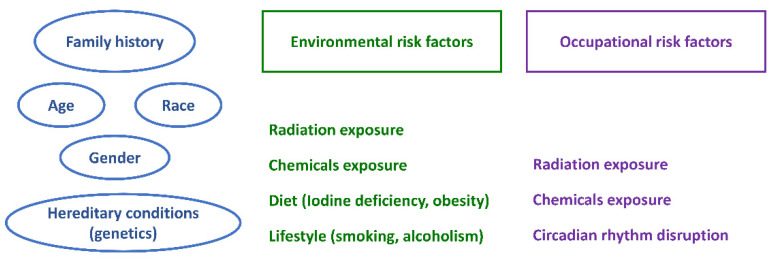
Main risk factors involved in the pathogenesis of thyroid cancer.

The risk factors for thyroid masses can be separated into two categories depending on the possibility of preventive intervention. There are risk factors that cannot be influenced, such as gender, age, or inherited conditions, and risk factors that can be influenced, such as hormonal treatments, extensive diagnostic procedures, and environmental and occupational hazards. Figure 1 shows the main risk factors involved in the pathogenesis of TC.

About 20% of MTC results from inherited genetic abnormalities (RET gene) known as familial medullary thyroid carcinoma. This condition can occur alone, or it can be associated with other endocrine tumors (most commonly pheochromocytoma), known as multiple endocrine neoplasia 2. For other subtypes of thyroid cancer, the genetic basis is not very clear, but it has been observed that higher rates of TC can occur among people with uncommon genetic conditions such as familial adenomatous polyposis, Cowden disease, or Carney complex type I [45].

According to the International Agency of Research on Cancer, the risk of TC is higher as the body mass index (BMI) increases, the risk of follicular and papillary subtypes is influenced by iodine in the diet and external ionizing radiation [2], but also by circadian disruption—especially in insulin-resistant patients [46].

Endocrine disrupting chemicals (EDCs) represent a broad group of molecules potentially involved in thyroid, breast, and prostate carcinogenesis, such as heavy metals, organochlorinated pesticides, industrial chemicals, plastics and plasticizers, fuels, and paints, that are present in diverse occupational settings and the environment as air pollutants from industrial sites, but also—some of them—in daily usage, such as household products (cosmetics, detergents) [47,48].

Aiming to prevent occupational diseases, occupational exposure limits (OELs) are set to prevent morbidity and mortality arising from inhalation of a singular or multiple workplace chemical. While OELs are useful, they are limited compared to more advanced assessment tools because they do not account for the complexity of the work and non-occupational environments [49]. 

As public health may be concerned with outdoor pollutants generated by different occupational settings, several regulatory public policies affecting risk assessment have emerged and cumulative effects have been defined [50].

Recently, risk assessment has been greatly improved due to scientific and technological advances that have provided the opportunity to characterize both the contribution and interactions of multiple exposures and their effect on human health. Occupational and environmental hazards can have different impacts on human health, also depending on various individual factors such as socioeconomic or physiological status. In an attempt to address these particularities, a new concept named “exposome” was introduced. The exposome can be defined as the measure of all the exposures of an individual in a lifetime and how those exposures relate to his health. Exposomics is the study of the exposome and is based on very complex methods of internal and external exposure assessment [51].

### 5.1. Occupational Risk Factors

Thyroid diseases cause a variety of physical and mental symptoms that can influence a patient’s biological function, emotional well-being, and social life. However, their impact on workplace performance is still unknown. As a result, the goal of this research is to look at how thyroid diseases can affect patients’ occupational outcomes, such as their employment rate, sick leave, working ability, and income. Recently, a systematic review of Pubmed, Scopus, and Web of Knowledge databases was conducted on this topic [52].

Depending on the severity of the disease, patients diagnosed with hyperthyroidism or hypothyroidism had a higher risk of long-term sick leave than controls. In nearly a third of affected individuals, hyperthyroidism impairs working abilities—particularly in cases complicated by orbitopathy with diplopia. Thyroid illnesses may have an impact on many occupational outcomes, according to a recent study [52], but further research is needed to understand the association between specific pathological characteristics throughout time and workplace risk factors. This could entail complete, interdisciplinary thyroid care management, with advantages for patients’ work, social, and personal lives.

Occupational exposure to external risk factors relies on cumulative risk assessment and uses either direct reading instruments, laboratory-based analysis, or survey instruments such as job-exposure matrices calculated using high accuracy formulas based on detailed information regarding job history (industrial field, job description, and length of service) [53]. Modern occupational risk assessments should consider integrating risks from multiple exposure pathways and routes (evaluation of aggregate risk) and the combined risk from exposure to chemical and non-chemical stressors, emphasizing also the severity of toxic effects generated by interactive processes (cumulative risk) [53].

The results of a Nord American population-based case–control study detailed the necessary assessment steps for occupational exposures, related occupational groups, and the incidence of thyroid cancer, using a specific method of assessing occupational history [54].

An important aspect is represented by the correlation between thyroid dysfunction and occupational risk factors through work in alternating shifts—a significant risk involving changes in the level of thyroid hormones [55]. Some relevant studies on the relationship between the occupational environment and TC have also been reported [1,54,56]. Occupational exposure “meta-analysis” matrices were used to highlight the risk of malignant thyroid disease and to show the association between circadian abnormalities and thyroid pathology. Different jobs were coded according to the standard occupational classification manual (SOC) from 2018. Data has been supplemented with further information from the professional history throughout life—such as job title, activities or duties, company name, type of activity, and length of service. A recent study found correlations between thyroid dysfunction and work in alternating shifts and indicated a significant risk, reflected by significant changes in the level of thyroid hormones [55].

It is known that radiation exposure for personnel from diagnostic imaging and radiotherapy departments is also an occupational risk factor that may lead to the development of thyroid nodules. However, the correlation between occupational radiation exposure in hospitals and thyroid disease is not extensively recognized. A total of 1024 female nurses working in a single hospital and 2631 healthy female patients who had a health checkup at the same hospital during the same period were the subjects of a systematic study [57]. Thyroid nodules were found in 30.8% of nurses and 38.9% of female patients who had a screening check-up, while 10.4% of nurses and 7.6% of female patients had suspicious nodules that were further evaluated with ultrasonography-guided fine-needle aspiration and cytology. As a result of the evaluation, thyroid carcinoma was detected in 1.6% of nurses and 1.4% of female patients. Working in a hospital does not raise the risk of thyroid nodules or TC, according to the findings of this study.

The link between chronic occupational ionizing radiation exposure and TC in the medical field is poorly understood. Thyroid cancer incidence was assessed in a cohort study dealing with 90,245 radiology technologists who completed the baseline survey and were TC-free 2 years after certification, and 73,080 technologists who also completed a second questionnaire a decade later. Using survival analysis, researchers calculated the risk associated with working as a radiology technologist, taking into account the length of time spent working, the types of procedures performed, and work habits. The history of assisting patients for X-ray procedures at least 50 times was the only occupational history trait linked with prospectively detected TC. Length of service, expressed as years working as a radiology technologist in total, years performing diagnostic, therapeutic, and nuclear medicine operations and employment under the age of 20 did not have a significant associations with the risk of TC [58]. Medical personnel exposure to radiation has been linked to an elevated risk of thyroid cancer in the future. Long noncoding RNAs (lncRNAs) are critical regulators of cancer biology, but little is known about how they are expressed in TC tissues in radiation exposed patients. A recent study [59] focused on determining the differences in transcriptomes between TC tissues and adjacent healthy thyroid tissues. A total of 23 lncRNA and messenger RNA transcripts were found to be differentially expressed in the TC tissues using microarray technology and quantitative reverse transcription PCR, affecting many pathways including cysteine and methionine metabolism, propanoate metabolism, and carcinogenesis. This research offered a transcriptome-wide screening and analysis of the lncRNA expression profile in TC tissues from individuals who had medical occupational radiation exposure, laying the groundwork for future research into lncRNAs and TC formation and carcinogenic risk of medical personnel.

Although ionizing radiation exposure during childhood is a well-known risk factor for TC, the risk linked with adulthood exposure is unknown. A large prospective study in the US Radiologic Technologists cohort looked at the link between cumulative low-to-moderate dose occupational thyroid radiation exposure and TC incidence. At least two of the four mailed questions were completed by 89,897 study participants who were cancer-free at the time of the first questionnaire. Self-reported work history, historical data, and 783,000 individual film badge measurements from 1960 to 1997 were used to calculate the cumulative occupational thyroid radiation exposure (mean 557 mGy, range 50–1600 mGy). Using Poisson regression to determine the excess relative risk (ERR) of TC (ERR/100 mGy absorbed dose) during follow-up, 476 TC cases were identified. After controlling for age, gender, birth year, weight and cigarette pack-years smoked, no link between thyroid dose and TC risk was found [60].

A more recent study [61] was centered on the hypothesis that exposure to radiation, particularly during youth, increased the risk of TC, further considering that nothing is known about low-dose radiation’s long-term impact on the risk of TC in adults. The impact of radiation on TC rates, as well as a general assessment of TC risk among medical radiation workers, was investigated in this study. Between 1996 and 2011, data on all radiation-exposed professionals involved in diagnostic medicine and enrolled in the national dosimetry registry were collected and matched with cancer registry data up to 2015. Internal comparisons were used to calculate relative risks (RRs) for occupational history, and ERRs were used to quantify the radiation dose–response connection, while standardized incidence ratios (SIRs) were used to compare the observed cancer incidence rates in this sample to those in the general population. There were 827 TC cases documented out of a total of 93,922 medical radiation employees. The TC SIRs for both genders were significantly higher than expected (SIR 1.18, 95% CI 1.08 to 1.28). There was no consistent pattern in the RRs for TC by job title or duration of employment among medical radiation employees. There was no evidence of a significant dose-effect on TC rates in either males (ERR/100 mGy 0.07, 95% CI) and females (ERR/100 mGy −0.13, 95% CI). When the research was restricted to workers who had been employed for at least a year, the results were similar across a wide range of job titles.

While the rate of TC was higher among South Korean medical radiation workers than in the general population, there was no evidence that this was due to occupational radiation exposure [61]. 

Several studies were carried out to determine which activities and sectors had a greater prevalence of TC. In Connecticut (USA) for example, a case–control study with 462 histologically confirmed cases and 498 controls was performed between 2010 and 2011. Certain occupations have been linked to an increased incidence of thyroid cancer, depending on the subtype or size of the tumor. Papillary microcarcinoma was shown to be significantly more common in people who worked as healthcare practitioners, technical personnel, and registered nurses. Workers in the cleaning, maintenance, pest control, retail sales, and customer service industries had a higher risk of developing PTC (tumors larger than 1 cm), as well as cooks, janitors, cleaners, and customer service representatives [54].

Another study was carried out to determine which jobs and industries in Sweden were more prone to developing TC. For the years 1971–1988, normalized incidence ratios for each employment and industry were computed using record-linkage with the Swedish National Cancer and Death Registers. Relative risks were assessed using Poisson models and adjusted for age, time, and geographic location. Teachers, carpenters, police officers, detention center officials, employees of agricultural machinery manufacturing, computing or accessory fabrication, public administration, or police administration were all linked to a higher risk for men, whereas medical technicians, shop managers, tailors, shoe cutters, and employees in the manufacture of prefabricated wooden buildings, electric installation, and wholesale of live animals, fertilizers, oilseed, or grain were all linked to a higher risk for women. Additional research is needed to explore the influence of specific chemical agents produced in some of the above-mentioned work situations [62]. A case–control study published in 2009 tried to find convincing evidence linking endocrine disruptor chemicals to TC in connection to occupation, in order to provide significant insight into TC risk factors that can be avoided. Several workplace chemicals, such as organochlorine insecticides, polychlorinated biphenyl, polybrominated biphenyl ethers, and anions such as perchlorate and nitrate, have been reported to disrupt thyroid hormone metabolism and regulation, although their carcinogenic risk has yet to be confirmed [63]. 

### 5.2. Environmental Risk Factors

The connection between environmental risk elements and TC promotion was recently reviewed [64]. The association between differentiated TC risk and the energy-adjusted Dietary Inflammatory Index (E-DII) was examined in a population-based case–control study conducted in New Caledonia, a Pacific archipelago with one of the highest recorded TC incidence rates in the world, to investigate the potential role of diet-induced inflammation. Data from a food frequency questionnaire on usual dietary intake was used to determine the E-DII. Logistic regression was used to evaluate data from 324 histologically confirmed cases of PTC and FTC diagnosed between 1993 and 1999, as well as 402 controls. There were positive correlations between E-DII and TC risk, the larger carcinomas having stronger associations. In conclusion, a pro-inflammatory diet has been linked to an increased risk of TC, particularly when associated with other inflammatory disorders or habits (e.g., obesity, smoking) [65].

The combined action of several dietary and environmental risk factors could be involved in the pathogenesis of an important number of pathological conditions, including cancer, through fibrinolitic degradation. The authors recommend the study of protein hydrophobic interactions for cancers linked to the above-mentioned risk factors [66].

Thyroid parenchymal and hormone changes have been observed in animals exposed to mercury (Hg) in various chemical states. The tests did not allow the development of dose–response curves or the confirmation of whether Hg effects on the thyroid parenchyma occur in humans. The focus of a recent report was to see if there was a link between persistent occupational exposure to metallic Hg and alterations in thyroid hormone homeostasis and gland parenchyma 14 years after exposure [67]. In a cross-sectional study conducted in a Brazilian hospital from 2016 to 2017, 55 males who had previously been exposed to metallic Hg and 55 males who had never been exposed were paired by age. Total and free triiodothyronine (TT3 and FT3), free thyroxine (FT4), thyrotropin (TSH), reverse T3 (RT3), selenium, and antithyroid antibody levels, as well as urinary Hg and iodine levels, were all tested, and the thyroid parenchyma was evaluated using B-mode ultrasonography. The Bethesda system was used for the cytological analysis of nodules with suspicious elements of malignancy following the FNA procedure. Univariate and multivariate logistic regression models revealed that the Hg-exposed group had higher serum TSH concentrations and a higher prevalence of parenchymal alterations, even after cessation of exposure, and in this case, it was recommended that the thyroid status of exposed workers be followed for a long time [67]. The link between exposure to polycyclic aromatic hydrocarbons (PAHs) and thyroid diseases is uncertain. Using the GC–MS/MS approach, eleven common hydroxylated PAHs (OH-PAHs) were recently detected in the urine of thyroid nodular goiter (NG) patients, PTC patients, and healthy controls. The levels of 2-hydroxyfluorene, 2-hydroxydibenzofuran, and 1-hydroxyphenanthrene in the NG group were considerably greater than those in the controls, as were the levels of 2-hydroxynaphthalene, 2-hydroxydibenzofuran, and 1-hydroxyphenanthrene in the PTC group. These findings suggest that PAH exposure may increase the risk of NG/PTC and that PAH exposure may affect NG/PTC development differently depending on gender [68]. Over the past few decades, risk factor cohort research has been published. A cohort study of TC, enrolling 204,964 individuals (aged 10–89 at baseline in 1964–1973, 54% female) was tracked for a median of 20 years using data from a major health plan. Thyroid malignancies occurred in 196 people (73 in men, 123 in women). Female gender, Asian race, completed college or post-graduate education, history of goiter, neck irradiation, and family history of thyroid disease were all independently and positively related to risk; Black race was shown to have an inverse relationship. Cigarette smoking, alcohol intake, hyperthyroidism, hypothyroidism, overweight or obesity, weight gain since age 20, height, occupational exposure, reproductive aspects, oral contraceptives, and hormone use did not have statistically significant associations with TC. These findings support the hypothesis that female gender, radiation, goiter, Asian race, high academic performance, and a family history of thyroid illness all play a role in the etiology of TC [69]. Another cohort research work examined the incidence of TC among rescue and recovery workers who had been exposed to the World Trade Center (WTC) events and had symptomatic or asymptomatic thyroid cancer. Increased TC incidence rates among exposed individuals may be linked to asymptomatic malignancies due to medical surveillance. This study examines the link between WTC exposure and TC among the New York City Fire Department, as well as the link with medical surveillance. The technique of detection (asymptomatic and symptomatic) for thyroid tumors in 14,987 males tracked from 2001 to 2018 was classified in this closed-cohort study. Using age-standardized rates, relative rates (RRs), and 95 percent confidence intervals, age-, sex-, and histologic-specific incidence rates were estimated and compared with demographically similar men. Papillary carcinomas were the focus of the secondary analysis. There were 72 cases of TC in the Fire Department after 9/11. A total of 53 cases (81.5%) were asymptomatic, while 12 cases (18.5%) were symptomatic among the 65 cases (90.3%) with a defined detection method. A total of 53 cases (81.5%) were asymptomatic, while 12 cases (18.5%) were symptomatic among the 65 cases (90.3%) with a categorized detection method. For asymptomatic vs. symptomatic cases, the median (interquartile range) age at diagnosis was 50.2 (44.0–58.6) years vs. 46.6 (43.9–52.9) years. The presence of occult lesions during medical surveillance appears to be the cause of excess asymptomatic TC among Fire Department WTC-exposed rescue/recovery workers. These findings have substantial implications for how TC incidence rates are perceived and how diagnoses should be managed among WTC-exposed populations and the general population [70].

The demographic data and questionnaire responses of TC cases from the Mount Sinai WTC Health Program were used in a recent study to explore TC in World Trade Center (WTC) responders (WTCHP). When compared to a subset of TC cases treated at Mount Sinai without WTC exposure, WTCHP TC tumors were of similar size (*p* = 0.4) and detected at a similar age (*p* = 0.2). These findings contradict the monitoring bias hypothesis, which states that smaller tumors are detected at a younger age. WTCHP TC cases also reported lower-than-expected rates of previous cancer diagnoses, family history of other malignancies, and high BMIs, as well as a history of radiation exposure and a family history of thyroid disorders [71]. It was concluded that further research is needed for a better understanding of the risk factors’ impact and role in the development of TC in this group. 

Differentiated TC (DTC), particularly the follicular type, is a serious clinical condition influenced by significant environmental variables such as iodine deficit and subsequent supplementation, thyroiditis, and occupational exposure. The characteristics of DTC in the Italian population were assessed in 2012, 20 years after iodine-prophylaxis, to consider the consequences of the 2005 release of updated guidelines for DTC diagnosis and management.

Between 1997 and 2010, 322 patients, including 244 females, with an average age of 53.8 and a 20% employment rate in the textile industry, were treated for DTC in a tertiary care institution and retrospectively analyzed. Medical history, demographics, and pathological characteristics were all taken into account. Patients were categorized into two groups: A (*n* = 139, diagnosis 1997–2005) and B (*n* = 183, diagnosis 2006–2010). Lymph node metastases and/or a late diagnosis increased thyroglobulin levels at the first follow-up, and along with further radioiodine therapy were linked to recurrent or chronic illnesses. The researchers reached the conclusion that tumor-related inflammation and occupational exposure are important factors in the etiology of DTC, although more research is needed to corroborate these findings [72]. 

Other risk factors, such as associations with reproductive problems, were investigated in a prospective study of 63,090 Norwegian women, with 124 cases of TC diagnosed from 1961 to 1989. No strong associations were found, but the risk of TC was significantly increased among women in the occupational category “fishing, ships officers and crew”. Finally, a modest effect of certain reproductive factors upon TC development was noticed [73].

Even though the frequency of TC has rapidly increased in South Korea over the last decade, few studies have looked into its risk factors. A new study investigated the factors that increase the incidence of TC in Korean individuals. A hospital-based case–control study was used in this investigation. There were 802 TC cases out of 34,211 patients between 2002 and 2011. Females and those with a family history of thyroid cancer had a higher risk of thyroid cancer, but never-smokers and those with a higher monthly household income had a lower risk of thyroid cancer, according to a multivariate conditional logistic regression analysis. A family history of cancer and alcohol intake was linked to a lower risk of thyroid cancer, but a higher BMI and a family history of thyroid cancer were linked to a higher risk of thyroid cancer. Females, those with a family history of thyroid cancer, those with a higher BMI, non-smokers, non-drinkers, and those with a lower monthly household income have a higher risk of developing TC, according to this data [74]. 

A meta-analysis assessed the association between obesity and TC risk. Data collected from PubMed, EMBASE, Springer Link, Ovid, and Chinese Databases retrieved before 2014 showed the values of adjusted risk ratios (RRs), hazard ratios (HRs), or odds ratios (ORs), and 95% confidence intervals (CIs) of TC risk. The 32 studies included in this meta-analysis showed that obesity was associated with a significantly increased risk of TC (adjusted RR = 1.33; 95% CI, 1.24–1.42; I^2^ = 25%). In cohort and case–control studies, an increased risk of TC was observed. When ethnicity was considered, Caucasians and Asians were shown to be at much higher risk. Both young and senior people had higher TC risk in the age subgroup study. Subgroup analysis based on smoking status revealed that both smokers and non-smokers had higher TC risk. Increased risk of PTC, FTC, and ATC were found in the histological subgroup analysis. As a result, obesity can be linked to an increased risk of TC, with the exception of MTC, where the results are still unclear [75].

## 6. Risk Factors and Risk Assessments: An Integrative Approach

Occupational risk assessment (RA) is a method for estimating health risks generated from exposure to various levels of workplace hazards. The aim of RA is to be able to anticipate a potential negative outcome, the probability of the event, and the subsequent general consequences that can arise. It is important to understand how much exposure to a hazard influences the health of workers to appropriately eliminate, control, and reduce those specific risks. New technologies with new or complex risks represent a constant challenge for occupational health specialists, with implications for future workforce safety and health [76].

Identifying specific occupational carcinogens (chemical and non-chemical) is an important and challenging topic with high relevance to science and public health, potentially providing the scientific basis for official recommendations. A recent study proposed an interesting design for identifying occupations at risk for TC. The authors used a standardized, structured questionnaire to collect information on lifetime occupational history and other suspected risk factors for TC, using the Standard Occupational Classification (SOC) system for job coding [54,77].

The risk attributable to early-life exposures (childhood, adolescence, and even pre-conception exposures for both parents) can contribute to effects at any time later in life. Applying an exposome-wide association study (ExWAS) approach in TC research allows for the development of rigorous computational models and tools for analyzing the dynamic and complex interactions between genetics, epigenetics, exposomics factors, and the omics profile [78,79].

## 7. Conclusions

Thyroid cancer diagnostics related to occupational and environmental risk factors require a multi-disciplinary approach and the involvement of biochemists, geneticists, physicians (oncologists, surgeons, endocrinologists, occupational health specialists), and statisticians for a better understanding of tumor development, accurate diagnosis by specific biomarkers, and the need for early intervention through targeted preventive actions.

The identification of specific biomarkers for thyroid cancer needs to be correlated with a Risk Assessment Matrix which addresses different key aspects of occupational and non-occupational risk factors, via complex questionnaires for data collection.

The occupational environment should be described in a very accurate manner, taking into consideration the current workplace, as well as all previous jobs and data regarding risk factors such as age, gender, family history, lifestyle habits, use of chemicals, or radiation exposure outside the workplace. A complex experimental design is needed, containing detailed working protocols to achieve a coherent and sustainable well-defined research pathway from sample collection to the identification of biomarkers, with correlations between specific oncometabolites integrated into Risk Assessment Matrices.

## Figures and Tables

**Table 1:** Comparative significance of risk categories for current TI-RADS assessment systems (ACR—American College of Radiology [18], EU—European [19] and K—Korean [20]) and the recommendations concerning TN threshold sizes for fine needle aspiration (FNA) and follow-up.

TI-RADS Category	Meaning	Risk of Malignancy	Size Threshold for FNA (cm)	Size Threshold for Follow-Up (cm)
TR 1	Benign (ACR) No nodule (EU, K)	-	No FNA	No follow-up
TR 2	Not suspicious (ACR) Benign (EU, K)	<2% (ACR) 0% (EU) <3% (K)	No FNA	No follow-up
TR 3	Mildly suspicious (ACR) Low risk (EU, K)	2–5% (ACR) 2–4% (EU) 3–15% (K)	>2.5 (ACR) >2 (EU) >1.5 (K)	>1.5 (ACR) >1 (EU)
TR 4	Moderately suspicious (ACR) Intermediate risk (EU, K)	5–20% (ACR) 6–17% (EU) 15–50% (K)	>1.5 (ACR) >1.5 (EU) >1 (K)	>1 (ACR) >0.5 (EU)
TR 5	Highly suspicious (ACR) High risk (EU, K)	>20% (ACR) 26–87% (EU) >60% (K)	>1 (ACR) >1 (EU) >1 (K)	>0.5 (ACR) All (EU)

## Data Availability

Not applicable.
